# Medical News

**Published:** 1856-02

**Authors:** 


### THE MEDICAL EXAMINER.

PHIADLELPHIA, FEBRUARY, 1856.

### MEDICAL NEWS.

As the coincidence of scarlet fever with variolous disease is rarely seen, a short account of a case in which it was observed may not be uninteresting.

Elizabeth ——, aged seven years, vaccinated when an infant, returned from her morning school on Wednesday, the 23d of January, complaining of feeling unwell, a pain in her chest, &c. Soon afterwards she had a chill, which was followed by high and even violent fever. At half past four in the afternoon I was summoned to see her in a convulsion.

On the next morning I found her face and chest covered with the rash of scarlet fever.

On Friday the eruption was bright and intense, and covered the whole body. Fever still high.

On Saturday the eruption was not so decided upon the face and chest, though still very bright upon the abdomen and lower limbs. Patient better.

On visiting her on Sunday morning I was informed by her mother that her fever had much increased during the previous evening, that she had been slightly delirious in the night, and that another eruption had made its appearance upon her neck and arms. Upon examination I found these to consist of the characteristic papules of variola.

On Monday the papules were both more numerous and larger. Scarlatinous rash still well marked upon the back.

I should mention that she had been exposed some time previous to the commencement of her sickness to the contagion of both diseases, and that there was during the whole continuance of it, another case of scarlet fever in the same house.

That two diseases differing so widely in their invasion, symptoms, progress, duration and peculiar eruption should be simultaneously developed in the system and run their course, independently of and apparently unaltered by each other, is certainly remarkable.

If any of our readers have met with a similar case or cases, I should be pleased to hear of them.—EDITOR.

---

A few days since we received an article for the Examiner from Prof. A. Hall, of McGill College, Montreal, in which a statement made by the editor of the "Northern Lancet," Dr. Horace Nelson, in his notice of Dr. Gross's work on Diseases of the Urinary Organs is severely criticised. In the appendix to Dr. Gross's work, Dr. Hall's name is given as authority for the assertion that calculous diseases are extremely rare in Montreal and its neighborhood, particularly among the French Canadians. This statement, which was denied and insultingly ridiculed by Dr. Nelson in his Journal, is reasserted in the most positive manner in the article sent us by Dr. Hall, under the authority of Prof. Holmes, Dr. Beaubien, Prof. Campbell, Dr. Belin, Dr. Johnston, Dr. Wight and Dr. Sheriff. The statistics of the two Hospitals, the Montreal General Hospital and the Hotel Dieu, are also given in evidence. Regretting that our limits will not permit us to publish Dr. Hall's paper, we refer such of our readers as are desirous of more information upon the subject, to Dr. Hall's paper in the next (February) number of the Montreal Medical Chronicle.

---

We had intended to have extracted a paper from the Boston Medical and Surgical Journal, (January 3d), containing the remarks of several physicians before the Boston Society for Medical Improvement, upon a case of Death from the "Symptomatic Vomiting of Pregnancy, &c.," related by Dr. J. B. S. Jackson, but were obliged to omit it for want of space. The following, from a part of Dr. Morland's subsequent researches upon the subject, will be found interesting.

"From the recent researches of M. Cartaya, of Cuba, the following results have been derived. 'In 58 examples of intractable vomiting during pregnancy' there were '30 cases of death, and 28 of recovery. Of these 28 recoveries, 11 were ascribed to the occurrence of abortion or the death of the fœtus and its subsequent expulsion; 2 to the influence of therapeutical remedies; and 1 to the occurrence of critical diarrhœa; 14 to the artificial induction of premature labor.

"'The principal object in adducing these facts, is to draw attention to the important nature of the affection to which they relate, and the resistance it offers to the therapeutical remedies hitherto employed in its treatment; and to impress upon medical men the necessity of multiplying those means and resources which observation and experiment may suggest as applicable to the difficulties arising from this serious complication in utero-gestation.' (*Loc. cit.*)

"As will be seen by the above enumeration of M. Cartaya, more than half the cases cited were fatal; the occurrence of premature labor, induced or accidental, was followed by cure in nearly half of the reported recoveries, and this fact speaks loudly in favor of the practice. As Dr. Churchill remarks, in speaking of the fatal case above referred to as occurring to Dr. Johnson, so in all critical cases, 'surely the induction of premature labor' would be not only 'justifiable,' but imperatively demanded, as doubtless the only measure 'affording the mother a chance of recovery.'"

---

To the Medical Ppofession of the State of Pennsylvania.

The undersigned were appointed a Committee by the State Medical Society at its late session at Hollidaysburg, to address the Profession of the State of Pennsylvania, on the importance of a more thorough organization of the Profession throughout the State.

The undersigned, in the performance of this duty, beg leave earnestly and urgently to appeal to their professional brethren for a united and vigorous effort, in furtherance of this great object. The many advantages, which have resulted to science and to the profession, from the partial organization already effected, must be apparent. The published Transactions of the State Medical Society, since its foundation in 1850, are replete with valuable statistical information, which could not have been collected or made available, but for the concerted action developed by the County Medical Societies. From the systematized observation thus begun, if continued and extended, the most important results maybe anticipated, in reference to the history and character of the epidemics of your section of country, to meteorological and geological influences upon their nature and progress, and to improvements in our methods of treating and preventing them. No physician can stand aloof from co-operation in these great objects, without failing in his obligations to his profession, to the community, and to himself.

The good effects of *association* upon professional harmony are no less marked. The friendly relations elicited by personal intercourse in the County, State, and National organizations, soften the asperities which are occasionally engendered by professional rivalry, and exert a liberalizing and refining influence upon the feelings. In *association*, too, the educated physician derives support and protection from the encroachments of irregular practice. The establishment of medical societies in the different counties—to which those only are known to be admissible, who can maintain a high professional and social standing—cannot fail to affect the public estimate of practitioners of medicine. Membership will be recognized as a certificate of attainments and character; and the line of separation which is drawn by the profession will be acquiesced in by the public.

The effective organization of the profession demands not only the the co-operation of its members in the County Societies, but also attendance, in due rotation, at the Meetings of the State Society and National Association. In responding to the claims which may be thus occasionally made upon their time and means, the delegates from the County Societies will, it is believed, find ample personal compensation in opportunities of extended intercourse with the profession of the State and Union, and in the agreeable social meetings, which form so pleasant a feature on such occasions.

May it not be hoped, that the members of the profession throughout the State, who have not yet taken a part in its organization, will respond to the appeal now made to them? In those counties, in which Societies have not yet been established, the efforts of a few—of even two or three—may accomplish the desired object. The proper preliminary steps are a public call for a meeting of the regular practitioners of the County, and the adoption subsequently of a Constitution and By-Laws, to be submitted for approval to the Censors of the District. The Corresponding Secretary of the State Medical Society, Dr. T. H. Yardley, of Philadelphia, will furnish any further information on the subject.

J. H. GEMMILL,

J. B. BIDDLE,

J. N. WYTHES. } Committee.

---

At the Annual Meeting of the Philadelphia Northern Medical Association held January 11th, 1856, the following Officers were duly elected:

*President.*—Dr. J. F. Lamb.
*Vice President.*—Dr. Joseph R. Bryan.
*Treasurer.*—Dr. J. Henry Smaltz.
*Recording Secretary.*—Dr. L. Curtis.
*Corresponding Secretary.*—Dr. Wm. Mayburry.
*Reporting Secretary.*—Drs. D. M. Hart, and J. S. Heill.
*Counsellors.*—Drs. J. Rhein, N. L. Hatfield, A. Helffenstein, and Geo. J. Ziegler.
*Delegates to the American Medical Association.*—Drs. N. L. Hatfield, Townsend, J. F. Lamb, and J. H. Smaltz.

L. CURTIS, Rec. Sec.

### PHILADELPHIA COUNTY MEDICAL SOCIETY.

The following *Officers* and *Delegates* for 1856 were elected January 16th, 1856.

*President.*—Wilson Jewell, M. D.

*Vice-Presidents.*—George W. Norris, M. D., William N. Johnson, M. D.

*Recording Secretary.*—Anthony E. Stocker, M. D.

*Assistant Secretary.*—James A. Meigs, M. D.

*Corresponding Secretary.*—Alfred L. Kennedy, M. D.

*Treasurer.*—William Byrd Page, M. D.

*Censors.*—Thomas F. Betton, M. D., John B. Biddle, M. D., D. Francis Condie, M. D., Samuel Lewis, M. D., Lewis Rodman, M. D.

*Delegates to the American Medical Association.*

| | | |
|---|---|---|
| Thomas F. Betton, M. D., | A. Helffenstein, M. D., | Samuel Lewis, M. D., |
| D. Francis Condie, M. D., | Benj. S. Janney, M D., | John F. Lamb, M. D., |
| G. Emerson, M. D., | Prof. S. Jackson, M. D., | Richard J. Levis, M. D., |
| James V. Emlen, M. D., | Wilson Jewell, M. D., | Wm. Mayburry, M. D., |
| Edw. Hartshorne, M. D., | Wm. N. Johnson, M. D., | Lewis Rodman, M. D., |
| Isaac Hays, M. D., | Alfred L. Kennedy, M. D., | Francis West, M. D., |
| S. L. Hollingsworth, M. D., | Rene La Roche, M. D., | Caspar Wistar, M. D. |

*Delegates to the State Medical Society.*

| | | |
|---|---|---|
| James Ash, M. D., | Henry Hartshorne, M. D., | Wm. Mayburry, M. D., |
| Franklin Bache, M. D., | S. L. Hollingsworth, M. D., | Charles D. Meigs, M. D., |
| T. Hewson Bache, M. D., | Nath. L. Hatfield, M. D., | J. H. B. McClellan, M. D., |
| John Bell, M. D., | Edw. Hartshorne, M. D., | Arnold Naudain, M. D., |
| John B. Biddle, M. D., | Prof. S. Jackson, M. D., | John Neill, M. D., |
| Thomas F. Betton, M. D., | Samuel Jackson, M. D., | George W. Norris, M. D., |
| Joseph Carson, M. D., | Benj. S. Janney, M. D., | John M. Pugh, M. D., |
| Benj. H. Coates, M. D., | Wilson Jewell, M. D., | Lewis Rodman, M. D., |
| D. Francis Condie, M. D., | Edgar Janvier, M. D., | Isaac Remington, M. D., |
| G. Emerson, M. D., | W. L. Knight, M. D., | Henry Y. Smith, M. D., |
| Robert A. Given, M. D., | W. H. Klapp, M. D., | J. Henry Smaltz, M. D., |
| Lewis P. Gebhard, M. D., | R. S. Kenderdine, M. D., | George B. Wood, M. D., |
| Wm. W. Gerhard, M. D., | E. F. Leake, M. D., | Thos. H. Yardley, M. D., |
| Paul B. Goddard, M, D., | R. La Roche, M. D., | Jacob S. Zorns, M. D. |
| John D. Griscom, M. D., | | |

ANTHONY E. STOCKER, M. D.,
Recording Secretary, 443 Walnut st.

